# Professor Virchow

**Published:** 1898-10-08

**Authors:** 


Vol. XXV.—No. 628.] Edited by Sir Henry Burdett, K.C.B., and Solomon C. Smith, M.D., M.R.C.P. [Oct. 8, 1898.

### PROFESSOR VIRCHOW.

The reception afforded to Professor Virchow at St. Martin's Town Hall on Monday, as well as at the banquet given in his honour on Wednesday evening, were such as to furnish abundant evidence, if, indeed, any were needed, of the high esteem and respect with which the great pathologist is regarded in this country by all whose scientific education has enabled them to appreciate the extent and accuracy of his work. His great achievement—the reduction to order and system of the numerous and varied morbid growths which are included under the general term of tumours, and his classification of them with reference to the relations which they severally bear to adult or to embryonic structure, would alone be sufficient to establish his reputation as an observer and investigator of the first rank; and yet this achievement constitutes only one of his services to the healing art. His address on Monday, given as the second Huxley lecture, in commemoration of the great philosopher whose name it bears, was in itself a wonderful performance. Its effect was somewhat diminished as the Professor himself explained, by the circumstance that he had been unable or unwilling to compress what he had to say within the limits of time commonly observed in lecturing; and hence, at the very last, he was compelled to sacrifice continuity of thought and reasoning, in some passages, to the necessity of not greatly transcending the accustomed hour. Nevertheless, the lecture as delivered was a masterpiece of its kind; and the Professor promised his hearers an opportunity of reading it in the more complete form in which it was originally composed. Publication will be the more necessary, inasmuch as the voice could only have been carried to all parts of the room by efforts which a speaker of Professor Virchow's years could not reasonably be expected to make; while, although his enunciation of English was remarkably good, there were yet occasional deviations into German methods of pronunciation which rendered some words a little uncertain even to those who were not far removed from the platform. These were, of course, matters of necessity in the circumstances, and we doubt whether any Englishman of equal age could be found in the medical profession who would be able to address a German or a French audience in their own tongue with equal finish and success.

If we turn from the manner of the lecture to its matter, it was, as might be expected, an elaborate assertion, rather than a defence, of the author's well-known doctrines with regard to cellular pathology. Disease, he maintains, unless in the broadest sense parasitic, or produced by organisms introduced from without, depends upon perverted formation, or perverted activity, of the individual cells of which organs and organisms are built up, and which, and not the individual formed by them, are to be regarded as the actual depositories of life. The argument may perhaps be regarded as unassailable, which declares that, if a neoplasm be not an outgrowth of perverted cells of the tissue which surrounds it, it must be an example of the origin of living from non-living matter. The diseases which are parasitic, such, for instance, as are produced by the invasion of the tissues by pathogenetic bacteria, are still only the consequences of a poisonous action exerted by the secretions of those bacteria upon cells; but no reference was made, as the lecture was delivered, to the form of individual cell action which recent researches have brought into such prominence and invested with such importance, the resistance offered to invading bacteria by phagocytes. On this, and on many other considerations which would necessarily flow from the main subject, there was no time to discourse; but not the least merit of the address was its suggestiveness, and the numerous topics which it was calculated to bring before the minds alike of those who received and of those who were in some degree of opposition to its teaching. For, after all, there are still persons who think it possible that the blood plasma may have some share in the production of morbid changes, if only by modifying or determining the character of cell activities, or by reason of a proneness to be modified by them in its turn. At present, the problem is insoluble, like that of the respective claims to priority of the fowl and the egg. Its solution will be arrived at, if ever, by observation and experiment, but not by dogmatic pronouncement.

Few parts of the lecture were more charming than those in which it touched on personal topics. There was a certain tenderness in the Professor's reference to the beauty of the English custom of days of remembrance, to the influence upon Huxley's mind of the years of sea life when, freed from the formalism of the schools, thrown upon the use of his own intellect, compelled to test each single object as regards properties and history, he forgot the dogmas of the prevailing system, and became, first, a sceptic, and then an investigator. But the veritable triumph was when the Professor, after referring to Lord Lister as one of the greatest benefactors of the human race, turned round, amid the ringing cheers of the audience, to shake his old friend warmly by the hand, and thus to claim, as it were, an universal brotherhood for the creators and the exponents of science.

